# Rouviere’s Sulcus Analysis: A Critical Safety Analysis and a Guide to Safe Laparoscopic Cholecystectomy

**DOI:** 10.7759/cureus.39385

**Published:** 2023-05-23

**Authors:** Saurabh Sharma, Rajan Sood, Abhinav Garg, Sameer Anand

**Affiliations:** 1 Department of General Surgery, Maharishi Markandeshwar Medical College and Hospital, Solan, IND; 2 Department of Surgery, Maharishi Markandeshwar Medical College and Hospital, Solan, IND

**Keywords:** general surgery, bile duct, calots, rouviere sulcus, laparoscopic surgery

## Abstract

Introduction: Laparoscopic cholecystectomy (LC) is the most frequent surgical operation in general surgery. The focus of recent research has been on improving the procedure's safety. Over 80% of healthy livers have Rouviere's sulcus (RVS), which is a natural notch in the right lobe that is present in proximity to the confluence of the bile duct. It is frequently considered an important component of safety during LC. RVS demarcates the area of the common bile duct (CBD) from the liver bed for the gall bladder. This research intends to evaluate the frequency, its relation to CBD, and the critical view of safety (CVS) during LC.

Materials and Methods: An observational study was performed in a cohort of 50 patients listed for LC between September 2021 and September 2022. The presence of RVS was confirmed after liver retraction and dissection commenced. After the creation of CVS, its relationship with CBD was documented. Additionally, the position of the cystic lymph node was also documented during the dissection.

Results: The findings of this study revealed that out of 50 patients, only 40 (80%) had RVS. However, cystic lymph nodes were present more frequently in 48 (96%) patients. CVS was achieved in all the patients, and it revealed the presence of RVS above the cystic duct-CBD junction in 37 (74%), at the level of the junction in 11 (22%), and in two (4%) where the junction could not be demarcated.

Conclusion: RVS is a reliable marker to dissect laterally to CBD while doing LC, which does not require any dissection and can be appreciated early during the procedure. However, its presence along with the cystic lymph node gives a better anatomical understanding of the area of CBD, thereby assisting in conducting the procedure safely.

## Introduction

Safe laparoscopic surgery is augmented by a firm grasp of surgical anatomy. The gold standard procedure for symptomatic gallstones is laparoscopic cholecystectomy (LC); nevertheless, surgeons encounter an increased risk of bile duct injury. Laparoscopic surgery presents challenges in anatomical identification since the structures are visualized in two dimensions. In recent years, significant efforts have been made to reduce the risk of iatrogenic biliary tract injuries and complications during laparoscopic cholecystectomy [1]. Nearly 0.5% of laparoscopic cholecystectomies result in bile duct damage. Patient safety has become more essential in the recent decade; hence, it is paramount to determine markers representing the plane of the common bile duct (CBD) during dissection [2]. To avoid harming CBD, the dissection should be conducted lateral to CBD during LC. In recent accounts, Rouviere's sulcus has become a frequently mentioned landmark or reference point [3].

In 1924, a French surgeon named M.H. Rouviere first reported the fissure that now bears his name [1]. A sulcus of 2-5 centimeters in length, Rouviere's sulcus may be seen to the right of the liver hilum, above the caudate lobe. The right portal triad, or one of its segmental branches, is present within the sulcus. Cholangiographic investigations have shown that the sulcus reliably identifies the common bile duct's plane [4]. Identification is possible in as many as 80% of instances [5]. As a helpful LC anatomical landmark, Rouviere's sulcus was first identified in 1997 [6]. The pressure of carbon dioxide (CO2) insufflation widens Rouviere's sulcus, making it anatomically visible during laparoscopic cholecystectomy [3, 5].

The purpose of this study was to analyze 50 cases of laparoscopic cholecystectomy to observe the frequency of Rouviere's sulcus occurrence and check its reliability after attaining a critical view of safety by visualizing its relationship with the junction of the cystic duct and CBD.

## Materials and methods

A cohort of 50 consecutively selected patients undergoing LC between September 2021 and September 2022 was prospectively observed at Maharishi Markandeshwar Medical College and Hospital, Solan, Himachal Pradesh, India, after taking the Institutional Review Board's clearance (IEC number MMMCH/IEC/20/377) and with the consent of the study's participants. A thorough medical history was obtained, as was a physical examination with a full blood workup and abdominal ultrasound. The advantages and disadvantages of laparoscopic cholecystectomy were discussed with the patients. All surgeries were performed by a senior surgeon holding the rank of professor. Inclusion criteria included patients of both sexes, candidates for laparoscopic surgery, cholecystitis, and individuals older than 18. Children and adolescents with gall bladder empyema, gallbladder mucocele, cholecystitis chronic, cholecystitis contracta, and cholecystitis intrahepatic who were not candidates for laparoscopic surgery were excluded from the study.

All patients were given prophylactic antibiotics at the time of induction, and the procedure was performed under general anesthesia. A standard four-port cholecystectomy was performed with the creation of a pneumoperitoneum using a closed technique. The gall bladder was retracted towards the right shoulder, and the immediate presence of RVS was confirmed and documented. In a scenario where omental adhesions were present with the gall bladder, the presence of RVS was confirmed after adhesions were released. Posterior dissection commenced until the lower third of the gall bladder was dissected away from the liver bed. Anterior dissection was performed, and at this point, the location of the cystic lymph node was confirmed. After the CVS was achieved, the location of Rouviere’s sulcus at the junction of the cystic duct (CD) and CBD was noted. The CD and cystic arteries were clipped and cut and the gall bladder was dissected away from the liver bed. The gallbladder was extracted from the epigastric port.

Following the completion of the data collection, IBM® Statistical Package for Social Sciences (SPSS®) version 21.0 (IBM Corp., Armonk, NY, USA) was utilized. Parametric variables are expressed as n and percentage (%), and non-parametric variables as mean plus standard deviation. It was determined to be statistically significant if the p-value was less than 0.05.

## Results

Fifty patients having laparoscopic cholecystectomy were part of the prospective research. Based on this information, we observed that the age range of the study group ranged from 21 to 75 years of age, with the highest concentration of cases occurring between the ages of 30 and 40 years. (Table 1).

**Table 1:** Age groups of the patients

Age	Number
20 to 30 years	10
30 to 40 years	16
40 to 50 years	12
>50 years	12
Total	50

Eighty-two percent of the 50 cases had a body mass index (BMI) of 30 or more, making them obese (Table 2).

**Table 2:** BMI of the individuals

BMI	Frequency	Percentage	Cumulative percentage	Valid percentage
26.0	4	8.0	8.0	8.0
27.0	6	12.0	20.0	12.0
28.0	1	2.0	22.0	2.0
31.0	9	18.0	40.0	18.0
32.0	11	22.0	62.0	22.0
33.0	7	14.0	76.0	14.0
34.0	8	16.0	92.0	16.0
36.0	3	6.0	98.0	6.0
40.0	1	2.0	100.0	2.0
Total	50	100.0		100.0

**Figure 1:**
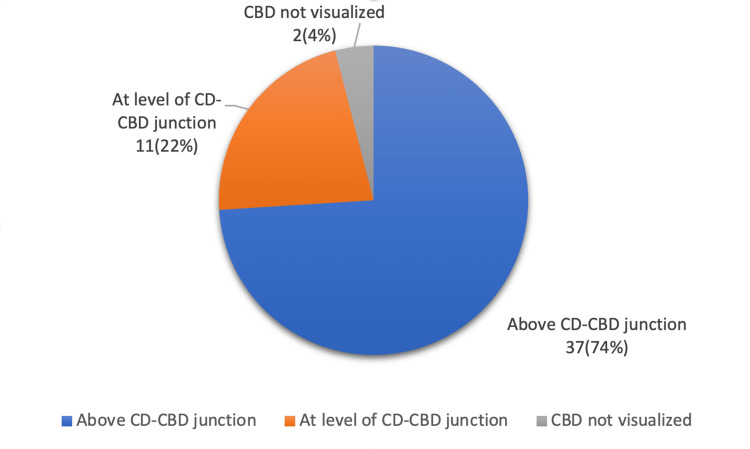
The presence of Rouviere's sulcus in relation to the common bile duct

The male-to-female ratio was 2.33, with 35 women and 15 men involved. Sixty-six percent of patients in our research were diagnosed with asymptomatic cholelithiasis, whereas 34 percent had chronic cholecystitis. Our research shows that 66% of the 50 instances included numerous gallbladder (GB) calculi, whereas 24 percent involved a single GB calculus. Forty of the patients in our research had RVS, whereas 10 did not. RVS was found in 40 people, with 26 of them having the closed type and 14 having the open type. After achieving the critical view of safety (CVS), the maximum number of patients had RVS above the level of the junction of CD and CBD, as explained in Table 3 and Figure 1.

**Table 3:** Frequency of the area of Rouviere's sulcus in relation to the cystic duct (CD) and common bile duct (CBD) junction

S. No.	Rouviere's sulcus	Number of patients	Percentage
1	Above CD-CBD junction	37	74
2	At the level of CD-CBD junction	11	22
3	CBD not visualized	2	4
	Total	50	100

## Discussion

Laparoscopic cholecystectomy (LC) is a commonly performed procedure that can be performed as a daycare procedure. The dreadful complications, like CBD injury, associated with the procedure cause prolonged morbidity for the patient. Therefore, strategies augmenting the safety of procedures are of paramount importance. Applying the CVS strategy helps minimize CBD injury, but achieving CVS is not always possible. An extrabiliary anatomical landmark, like Rouviere’s sulcus, would thus assist in conducting LC safely. We examined the use and reliability of RVS as a marker of safety during LC. Most patients presented between 30 and 40 years of age (32%), which corresponds with the literature [4]. Female preponderance is observed probably because pregnancy and childbirth may have a role in the increased prevalence of biliary tract illness in females by causing bile stasis, which leads to weight gain and hypercholesteremia [5]. Obesity is a risk factor for cholelithiasis due to saturation of the bile; therefore, it was prominent in our cohort as well [6].

The incidence of RVS in the literature differs from population to population and ranges from 60% to 90% [7-9]. This is comparable with the results of our study as well. RVS is visualized when the liver is retracted upwards along with the gall bladder with a laparoscopic grasper. An advanced high-definition imaging system provides precise visualization of RVS and adjacent structures. Anatomically, the RVS demarcates the area of confluence of hepatic ducts and contains segmental branches of the right hepatic duct and right portal vein. Therefore, it is recommended not to dissect below the RVS to avoid bile duct injury. Additionally, anterior to the sulcus is the area of the cystic artery and CD [10]. Even with the presence of adhesions around Calot's triangle, they can still be visualized outside the area of dissection and can be referred to as remaining in the safe plane of dissection. Hugh et al. found that starting from the RVS as an extrabiliary fixed point for dissection was not only useful but also safe [11].

The cystic lymph node is an important landmark for the identification of cystic arteries, but the level of the lymph node also helps in the identification of areas of the CBD [12]. The lower margin of the cystic lymph node demarcates the area of CBD; therefore, dissection should be done above the area where the cystic lymph node is present. Literature confirms the presence of cystic lymph nodes in more than 90% of the patients [13]. The combination of RVS and cystic lymph nodes helped maintain the plane of dissection away from the CBD, which was an interesting finding of the study [14]. RVS and cystic lymph node were found to be reliable anatomical markers for safe dissection, but it should be emphasized that safety can only be achieved when the principles of CVS creation are followed properly, along with knowledge of biliary anatomy and its variations [15]. Intraoperative timeout and a checklist are also musts while performing LC to reduce misinterpretation of structure and select the right bailout strategy [16].

The study has limitations; being a self-funded single-center study limits the sample size; however, it's the first study to include populations from the Himalayan region. The role of RVS in safe LC will be better understood by follow-up studies carried out in more centers with a larger sample size. Due to the small sample size, there is a chance of unintentional selection bias. For further information, it is advised to conduct a large-scale intraoperative laparoscopic study.

## Conclusions

Rouviere's sulcus is an extrabiliary, easily accessible, and reliable anatomical marker for the prevention of injury to the common bile duct during laparoscopic cholecystectomy. Additional identification of cystic lymph nodes along with RVS decreases the chances of dissecting CBD. Therefore, it can be used as an adjunct with a critical view of safety strategy during laparoscopic cholecystectomy to preclude injuring bile ducts.
